# Telephone training to improve ECG quality in remote screening for atrial fibrillation

**DOI:** 10.1088/1361-6579/ad9798

**Published:** 2024-12-17

**Authors:** Kethaki Prathivadi Bhayankaram, Jonathan Mant, James Brimicombe, Andrew Dymond, Kate Williams, Peter H Charlton, on behalf of the SAFER authorship group

**Affiliations:** Department of Public Health and Primary Care, University of Cambridge, Cambridge, CB1 8RN, United Kingdom

**Keywords:** single-lead ECG, quality, telephone training, remote screening, mobile device, community-based ECG, atrial fibrillation

## Abstract

*Objective.* Self-recorded, single-lead electrocardiograms (ECGs) are increasingly used to diagnose arrhythmias. However, they can be of variable quality, affecting the reliability of interpretation. In this analysis of ECGs collected in atrial fibrillation screening studies, our aims were to: (i) determine the quality of ECGs when recorded unsupervised; and (ii) investigate whether telephone training improved ECG quality. *Approach.* Data was obtained from the Screening for Atrial Fibrillation with ECG to Reduce stroke programme, where participants recorded four single-lead ECG traces per day for three weeks using a handheld device. ECG quality was assessed by an automated algorithm, and participants who recorded >25% poor-quality ECGs from days 4–10 of screening were identified for training to improve ECG recording technique. Training was delivered when research team capacity permitted. *Main results.* 13 741 participants recorded 1127 264 ECGs, of which 41 288 (3.7%) were poor-quality. Most participants (51.5%) did not record any poor-quality ECGs. 1,088 (7.9%) participants met the threshold for training. Of these, 165 participants received training and 923 did not. The median proportion of poor-quality ECGs per participant on days 1–3 was 41.7 (27.3–50.0)% for those who received training and 33.3 (25.0–45.5)% for those who did not. On days 11–21, the median proportions of poor-quality ECGs per participant were significantly lower (*p* < 0.001) for those who received training, 17.8 (5.0–31.6)%, and those who did not, 14.0 (4.8–30.2)%. Comparing these groups, the mean (95% confidence interval) reduction in proportion of poor-quality ECGs from days 1–3 to days 11–21 was 20.2 (16.8–23.5)% in those who received training and 16.0 (14.7–17.3)% in those who did not (*p* = 0.396). *Significance.* Most participants achieved adequate quality ECGs. For those that did not, ECG quality improved over time regardless of whether they received telephone training. Telephone training may therefore not be required to achieve improvements in ECG quality during screening.

## 1. Introduction

The electrocardiogram (ECG) has been used as a diagnostic investigation for cardiovascular disease and arrhythmia since its invention by Willem Einthoven in 1901 (Barold 2003). Whilst the ECG was primarily developed as a diagnostic tool (Harris 2016), it has also been used as an effective screening tool for cardiovascular disease so that patients who are found to have disease can be treated in the early stages before they start presenting with symptoms (Bhatia and Dorian 2018). The development of artificial intelligence algorithms has potential to revolutionise ECG interpretation (Attia *et al* 2021) with possible applications in devices such as smartwatches.

The quality of the ECG trace is key in successful interpretation to ensure that patients receive the correct diagnosis (Zhang and Hou 2015, Hibbitt *et al* 2024). For instance, a higher ECG quality has been associated with an increased ability to detect atrial fibrillation (AF) (Pipilas *et al* 2023). ECG quality is particularly important when using handheld ECG devices. The quality of ECG recordings acquired using these devices can be lower than that of 12-lead ECGs (Khamis *et al* 2016) due to: the use of dry electrodes instead of gel electrodes; recording at the hands rather than the chest; holding the device incorrectly; and recording without clinical supervision. Three single hospital-based studies have explored the quality of single-lead ECG traces captured using the Alivecor, MyDiagnostick and Zenicor One ECG devices in Belgium, Denmark and Germany (Desteghe *et al* 2017, Poulsen *et al* 2017, Wegner *et al* 2020) with the aim of detecting AF. In these studies, the proportion of ECGs reported as insufficient quality for interpretation was moderately high, varying between 4% and 22% (Desteghe *et al* 2017, Poulsen *et al* 2017, Wegner *et al* 2020), but none of these studies have tested any intervention to improve ECG quality (Desteghe *et al* 2017, Poulsen *et al* 2017, Wegner *et al* 2020).

**Figure 1.**
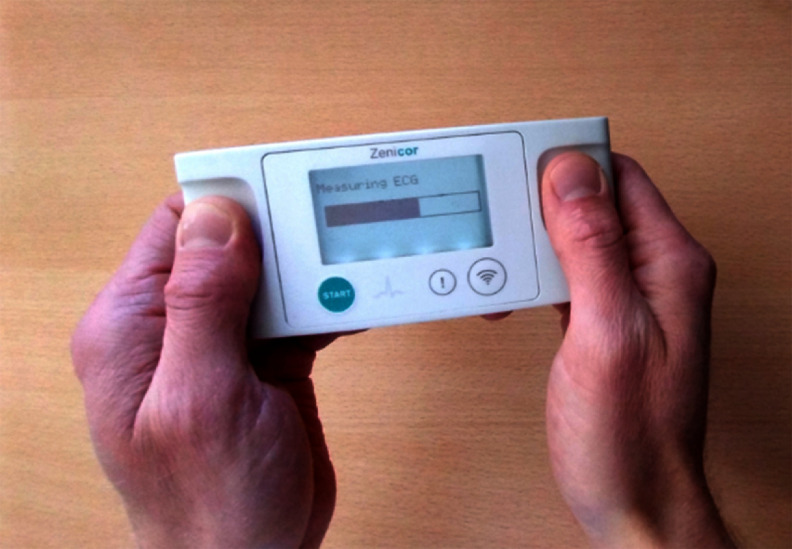
The Zenicor One ECG device used in the SAFER studies. Reproduced from Kristof *et al* (2024). CC BY 4.0.

The Screening for Atrial Fibrillation with ECG to Reduce stroke (SAFER) programme is being undertaken in the UK to assess the effectiveness of screening for AF to prevent stroke (Williams *et al* 2022). As part of this programme, participants are asked to record single-lead ECGs at home for 30 s four times a day. The hand-held single-lead ECG device used (Zenicor One, Zenicor Medical Systems AB, Sweden), shown in figure 1, can detect paroxysmal AF through screening, as demonstrated through a randomised controlled trial in Sweden (Svennberg *et al* 2021). The ECG traces are analysed by the ECG Parser algorithm (Cardiolund AB, Sweden) which is able to indicate possible abnormalities (Svennberg *et al* 2017). The algorithm is also able to detect whether an ECG trace is of adequate quality for accurate clinical interpretation or ‘poor-quality’ (i.e. the trace is of insufficient quality for accurate clinical interpretation).

One possible intervention to reduce the proportion of ‘poor-quality’ ECGs is the use of telephone calls to provide training in ECG recording technique. Telephone calls to provide training have been shown to improve adherence to a variety of interventions including exercise for cancer recovery (Mayer and Plumeau 2023) and continuous positive air pressure therapy (Schoch *et al* 2019). However, to our knowledge there is currently no published evidence exploring telephone calls as a possible intervention to improve self-recorded ECG quality.

Our first aim was to determine the quality of ECGs in participants who recorded them without any direct supervision (i.e. in their own homes). Our second aim was to determine whether telephone calls are effective in improving ECG quality. To investigate this, we identified participants who recorded a high proportion of poor-quality ECGs in the early stages of screening, and compared the quality of ECGs in the latter stages of screening between those who received additional telephone training and those who did not.

## 2. Methods

**Figure 2.**
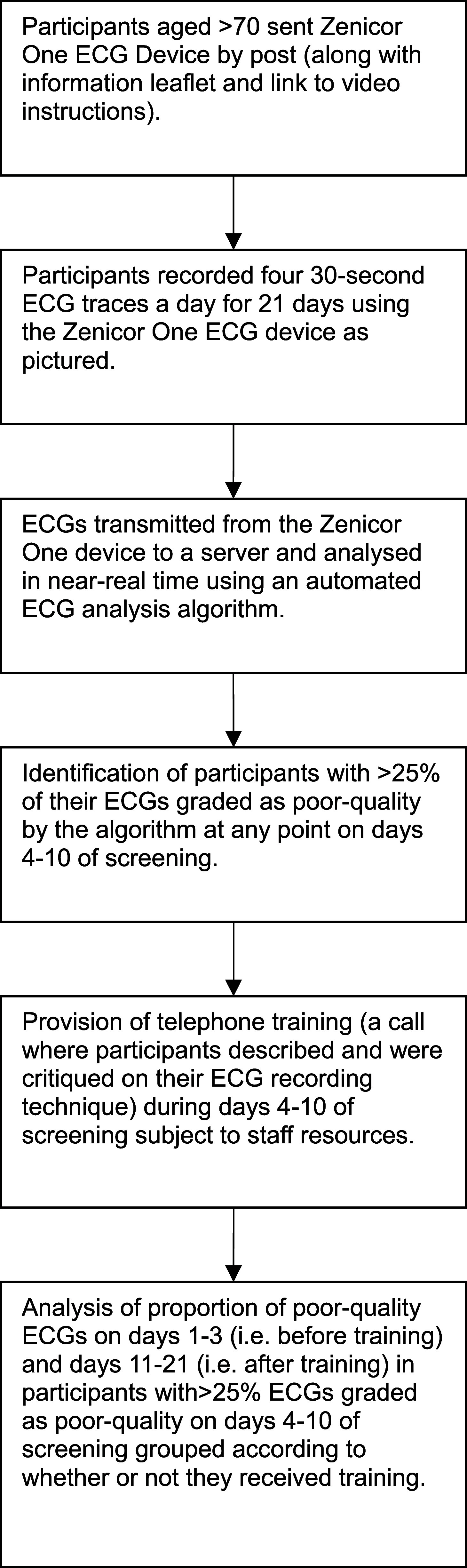
The of data collection and analysis processes.

Data was collected and analysed from the SAFER programme’s remote feasibility study (October 2020–January 2021) (ISRCTN 72104369, REC ref 19/LO/1597) and Trial (May 2021-present) (ISRCTN 72104369, REC ref 19/LO/1597). The Trial is still ongoing but in this study we used data collected up until April 2023. The data collection and analysis process is summarised in the text below and in figure 2.

**Data collection:** As part of the screening intervention, participants aged 70 and over were sent the Zenicor One ECG device by post with which to record a 30 s ECG four times per day for three weeks. Briefly, this device was used by participants to record single-lead ECGs between the thumbs using dry electrodes (as shown in figure 1). After each recording the user is asked to confirm that the ECG should be transmitted via mobile network to central servers. The device has an internal memory capable of storing ECGs should a mobile network not be available.

Participants were provided with written instructions on how to use the device which included a link to an online video summarising the process for recording an ECG and achieving high signal quality (https://vimeo.com/908283131). Some participants were randomly allocated to receive a call at the start of screening (to assess the value of such calls) during which they were informed about good ECG recording technique and asked to record an initial ECG. This initial ECG was reviewed for quality only, and if necessary further ECGs were recorded whilst on the call after reiterating the technique and adjusting if appropriate. This would be repeated until either a sufficiently high-quality ECG was recorded, or it was deemed that this would not be possible or the participant did not want to carry on. The screening duration was three weeks for all participants regardless of what their first ECG showed since ECGs were only analysed at the end of the three week screening period (Mant *et al* 2024).

Participants who might benefit from training telephone calls once the screening had started were identified as follows. ECGs were transmitted from the Zenicor One device to a server and analysed in near-real time using an automated ECG analysis algorithm (ECG Parser algorithm, Cardiolund AB, Sweden) (figure 2) (Svennberg *et al* 2017). Participants who had at least 25% of their ECGs graded as poor-quality by the algorithm at any point on days 4–10 of screening were identified, and the research team made a training telephone call to these participants where possible (figure 2). Here, days 4 and 10 were defined as the fourth and tenth calendar days of screening—*i.e.* if a participant recorded their first ECG on a Monday, then day 4 would be from 00:00 to 23:59 on the Thursday. Whether or not a training call was received was primarily due to logistical issues such as staff capacity and participant availability when the call was placed.

During the training call, each participant was asked to describe which recording technique they were using and where possible, guidance to improve technique was sensitively provided. If appropriate and possible, the participant recorded one or more further ECGs during the call, which if graded as poor-quality by the algorithm prompted additional feedback and/or guidance. The calls lasted an average (mean) of six minutes.

All participants gave informed consent, and the studies were conducted in accordance with the Declaration of Helsinki, and ethical approval was given by the London Central NHS Research Ethics Committee (18/LO/2066 and 19/LO/1597).

### 2.1. Data analysis

The dataset characteristics were reported in terms of the number of participants, number of ECGs and the number and proportion of participants with poor-quality ECGs at different thresholds (from 2% to 50%). Whether or not an ECG was tagged as ‘poor-quality’ was determined by the ECG Parser algorithm (Cardiolund AB, Sweden) (Svennberg *et al* 2017). Significant differences between the proportions of participants with at least one poor-quality ECG and the proportions of participants with at least 25% poor-quality ECGs were identified using the chi-square test.

Subsequent analyses focus on those participants who had at least 25% of their ECG traces tagged as ‘poor-quality’ by the automated algorithm at any time between days 4 and 10 of screening (figure 2). Since the data are non-parametric, the median, inter-quartile range and range of the proportion of poor-quality ECGs per participant were calculated for days 1–3 of this sub-group, and for days 11–21. We chose these time intervals because participants who received training were intended to receive it during days 4–10 (Williams *et al* 2022), and we excluded any participants who received training outside of this time period (i.e. before day 4 or after day 10 of screening). The data are presented as box and whisker plots. The Wilcoxon signed rank test was used to identify significant differences between the proportions of poor-quality ECGs per participant between days 1–3 and days 11–21.

We also compared the differences in the proportion of poor-quality ECGs between days 1–3 and days 11–21 between participants who received a training call and those who did not (figure 2). Since these differences are normally distributed, we calculated the mean difference and confidence intervals between the groups and used analysis of covariance (ANCOVA) to determine statistical significance between the intervention and control groups across both studies to ensure baseline standardisation (Vickers and Altman 2001). Due to the small number of participants in the remote feasibility study, we combined these data with the trial data for this analysis, thereby increasing the power available to detect a significant difference between groups.

To explore the possibility of regression toward the mean (i.e. outliers early in screening tending toward the average later in screening), we also compared the differences between days 1–3 and days 11–21 in people who recorded a low proportion of poor-quality ECGs (<5%) (Bland and Altman 1994). Again, results were reported as median, inter-quartile range and range, and significance testing was performed using the Wilcoxon signed rank test.

Analysis was performed using Microsoft Excel and MATLAB R2023a. The code used for the analysis is available to view online (Charlton 2024a, 2024b). All results are reported to one decimal place.

## 3. Results

### 3.1. Quality of ECG recordings

**Figure 3.**
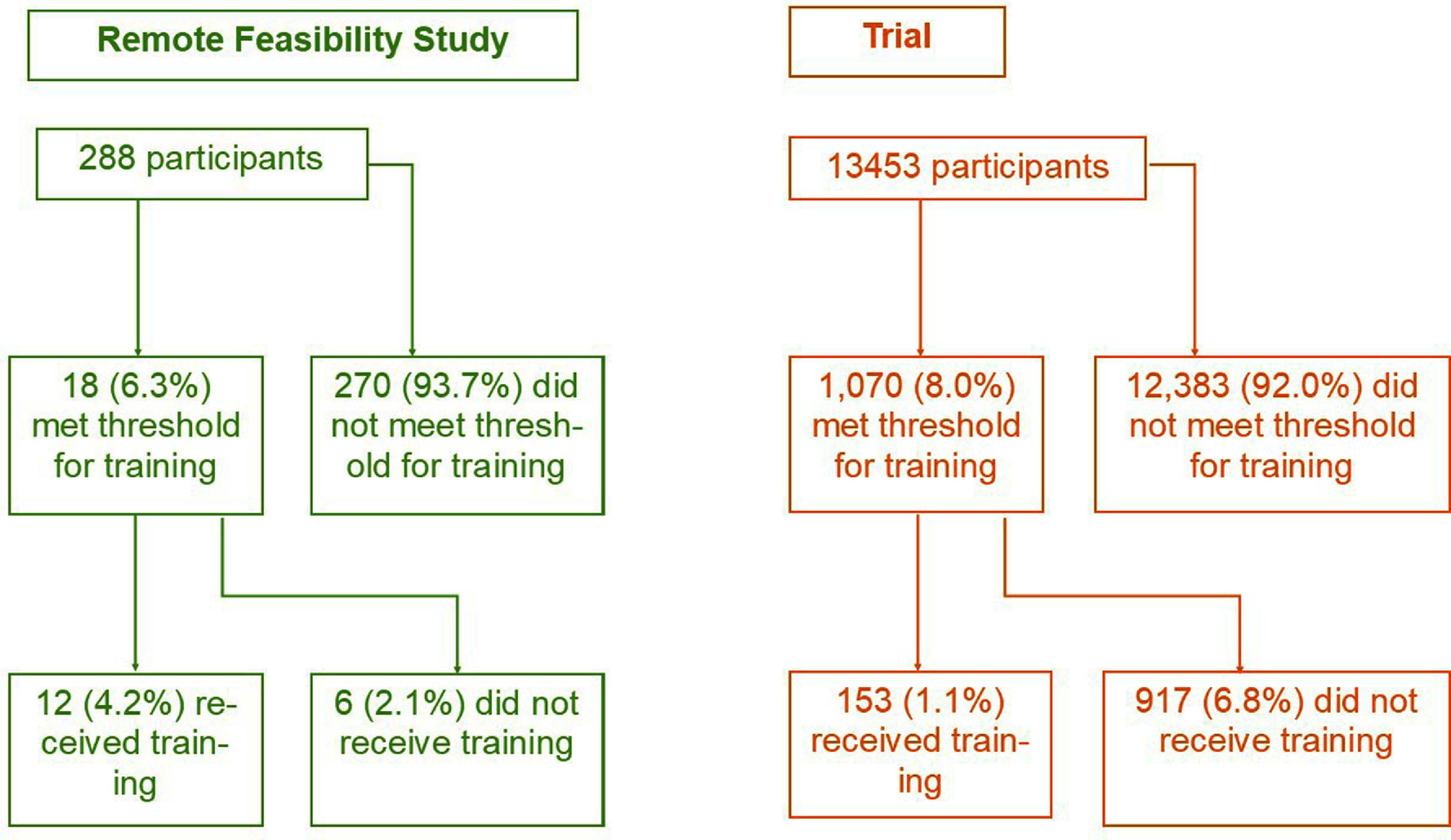
Flowchart illustrating the numbers and proportions of participants who met the threshold for a training call and subsequently either received a training call or did not receive one. The threshold for training was defined as having at least 25% of ECGs classified as ‘poor-quality’ at any time during days 4–10 of screening.

**Figure 4.**
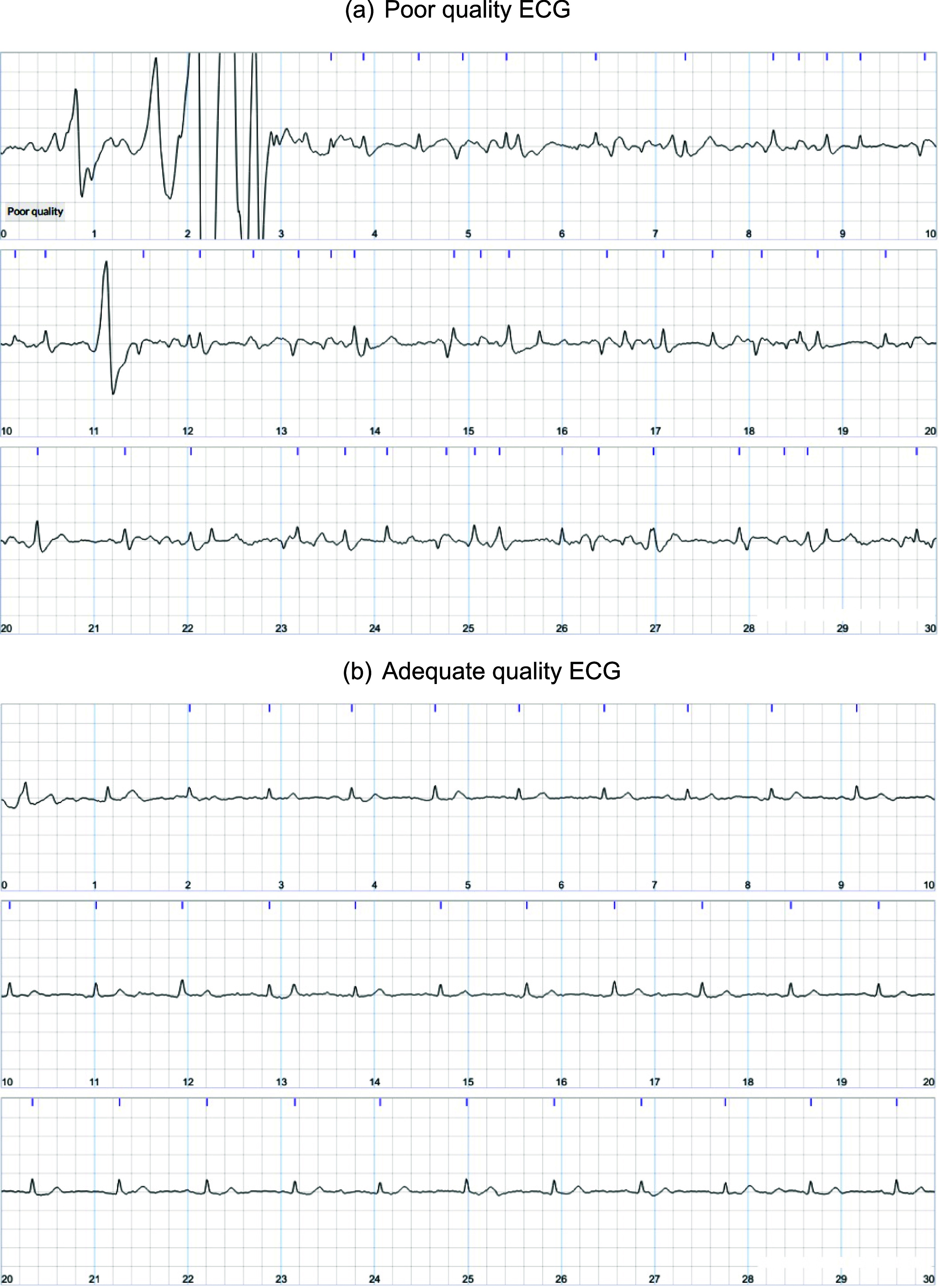
Example ECGs: (a) shows an ECG recorded by a trial participant on their first day of screening, which was tagged as ‘poor quality’ by the automated algorithm. It is difficult to reliably identify QRS complexes confidently in this ECG. (b) shows an ECG recorded by the same participant on their final day of screening, which was not tagged as ‘poor quality’, in which QRS complexes are clearly visible.

In summary, 13 741 participants from the SAFER remote feasibility study and trial were included in this analysis and together recorded 1127 264 ECGs, of which 41 288 (3.7%) were tagged as poor-quality (see table 1). Further information on the participant demographics, the number of participants who had poor-quality ECGs and the number of participants who met the threshold for and received training is provided in table 1 and figure 3. Figure 4 shows examples of poor-quality and adequate quality ECGs collected during screening.

**Table 1.** Characteristics of the SAFER remote feasibility study and trial datasets.

	Remote feasibility study	Trial	Total (remote feasibility study + trial)
No. participants	288	13 453	13 741
Age, mean (SD)	76.7 (5.9)	76.2 (4.8)	76.2 (4.9)
Gender, no (% female)	141 (49.0%)	6,904 (51.3%)	7,045 (51.3%)
No. GP practices	3	87	90
Total no. ECGs over 3 weeks	23 259	1104 005	1127 264
Total no. poor-quality ECGs (%) over 3 weeks	580 (2.5%)	40 708 (3.7%)	41 288 (3.7%)
No. (%) participants with at least one poor-quality ECG	123 (42.7%)	6,537 (48.6%)	6,660 (48.5%)
No. (%) participants with at least 25% poor-quality ECGs	5 (1.7%)	459 (3.4%)	464 (3.4%)
No. (%) participants who met the threshold for Training	18 (6.2%)	1,070 (8.0%)	1,088 (7.9%)
No. (%) participants who received training	12 (4.2%)	153 (1.1%)	165 (1.2%)

**Figure 5.**
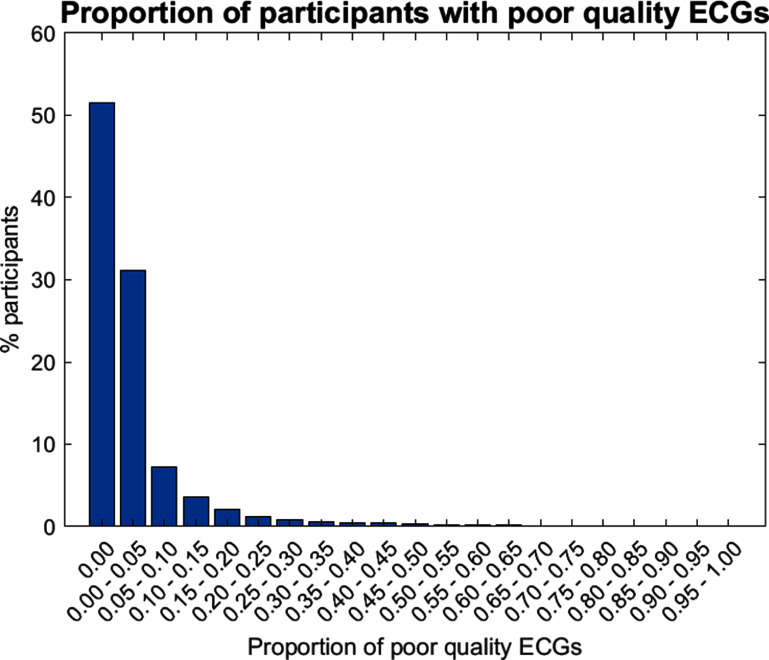
The proportions of participants with poor-quality ECGs at different thresholds in the SAFER programme. Note that the majority of participants did not have any poor-quality ECGs.

A higher proportion of participants had at least one ECG graded as poor-quality in the Trial as compared to the remote feasibility study (48.6% versus 42.7%, *p* < 0.0001, chi-squared = 190.073 with one degree of freedom) (see table 1). Similarly, there was a significant tendency for a higher proportion of participants in the trial to have at least 25% of their ECGs tagged as ‘poor-quality’ during screening (3.4% versus 1.7%, *p* < 0.0001, chi-squared = 220.176 with one degree of freedom) (see table 1). Figure 5 shows the distribution of proportions of poor-quality ECGs. The majority of participants had no poor-quality ECGs, approximately 30% of participants had between 0% and 5% poor-quality ECGs, and progressively fewer participants had higher proportions of poor-quality ECGs.

### 3.2. Effectiveness of training calls

**Figure 6.**
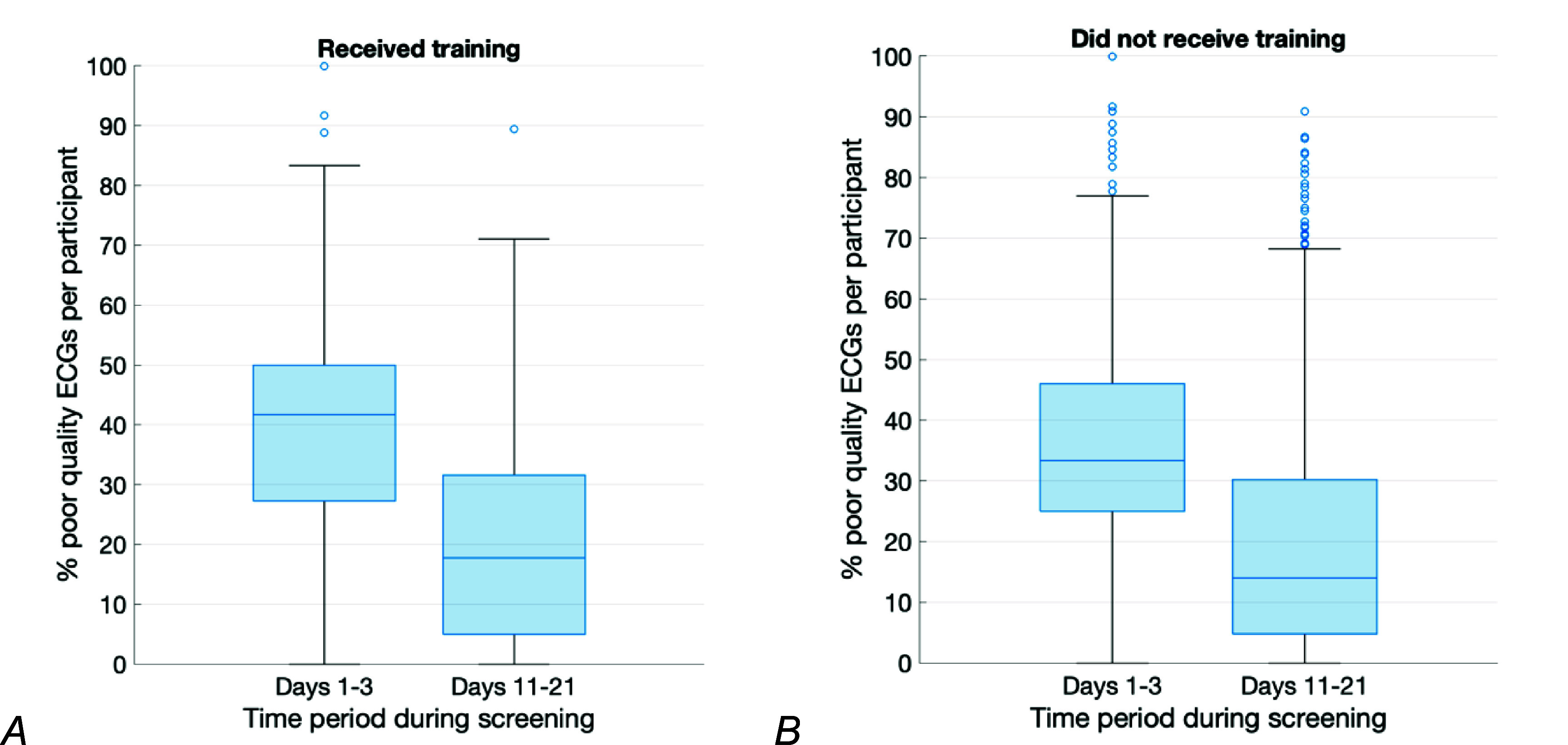
Box and whisker plots demonstrating the median and quartiles of proportions of poor-quality ECGs per participant on days 1–3 and 11–21 of screening in participants who met the threshold (>25% of their ECGs being marked as ‘poor-quality’ on days 4–10 of screening) for a training call and (A): received training and (B): did not receive training in the SAFER programme.

In the remote feasibility study, 18 participants (6.2%) met the threshold for training on days 4–10 of screening, of which 12 (4.2%) received training and 6 participants (2.1%) did not receive training (table 1, figure 3). In the trial, 1,070 participants (8.0%) met the threshold for training on days 4–10 of screening, of which 153 (1.1%) received a single training call and 917 participants (6.8%) did not receive training (table 1 and figure 3). Detailed analyses of the proportions of poor-quality ECGs per participant are shown in table 2 and figure 6.

**Table 2.** The proportions of poor-quality ECGs per participant on days 1–3 and days 11–21 of screening in participants who received training and participants who met the threshold but did not receive training.

	Median (IQR) proportion of poor-quality ECGs on days 1–3 of screening (%)	Median (IQR) proportion of poor-quality ECGs on days 11–21 (%)	*p*-value
*Received training*			

Remote feasibility study	30.0 (18.2–46.4)	5.1 (2.4–6.9)	0.016
Trial	41.7 (29.3–50.0)	18.2 (6.5–31.8)	<0.001
Combined	41.7 (27.3–50.0)	17.8 (5.0–31.6)	<0.001

*Did not receive training*			

Remote feasibility study	29.7 (21.4–33.3)	6.7 (0.0–18.6)	0.094
Trial	33.3 (25.0–46.2)	14.0 (4.9–30.3)	<0.001
Combined	33.3 (25.0–46.0)	14.0 (4.8–30.2)	<0.001

Combining the results from the remote feasibility study and the trial, the mean (95% confidence interval) difference between the proportions of poor-quality ECGs on days 1–3 and days 11–21 was 20.2 (16.8–23.5)% in the intervention group and 16.0 (14.7–17.3)% in the control group (table 3, figure 6). ANCOVA demonstrated that there was no significant difference (*p* = 0.396) in the changes in proportion of poor-quality ECGs between the participants who received telephone training and participants who met the threshold for telephone training but did not receive it.

**Table 3.** The differences between the proportions of poor-quality ECGs on days 1–3 and 11–21 in the remote feasibility study, trial and a combined analysis of the two, as well as the analysis of covariation (ANCOVA) result demonstrating the level of statistical significance between the differences.

	Mean (95% CI) difference between the proportions of poor-quality ECGs on days 1–3 and 11–21 (%)		
	Received training	Did not receive training	*p*-value
Remote feasibility study	23.2 (8.9–37.6)	19.1 (2.6–35.6)	0.649
Trial	19.9 (16.5–23.4)	16.0 (14.6–17.3)	0.668
Combined	20.2 (16.8–23.5)	16.0 (14.7–17.3)	0.396

Analysis of regression to the mean in participants with <5% poor-quality ECGs demonstrated that the median (IQR) proportion of poor-quality ECGs per participant was at 0 (0–0)% on days 1–3 and remained at 0 (0–0)% on days 11–21 (*p* > 0.05).

## 4. Discussion

### 4.1. Summary of findings

To the best of our knowledge, our study is the first to explore telephone training as a measure to improve the quality of ECGs in the context of remote screening for AF. We found that the majority of participants had sufficient high-quality ECGs to not require further training by telephone calls, in keeping with previous findings that 98% of participants in the SAFER programme had at least 56 interpretable ECGs from the screening period (Mant *et al* 2024). Amongst the small minority of participants who recorded high numbers of poor-quality ECGs during the initial days of screening, the quality of ECGs improved over time regardless of whether or not they received telephone training.

### 4.2. Comparison with existing literature

We found that 40%–50% of participants across the remote feasibility study and trial had at least one poor-quality ECG and 1%–3% had at least 25% of their ECGs marked as poor-quality. Comparable data in the context of AF screening have not been reported in the existing literature. However, one study has reported that a higher ECG quality is associated with a higher ability to detect AF, so strategies to improve the ECG quality will therefore improve AF detection (Pipilas *et al* 2023).

Participants across the two studies had 2%–4% of their ECGs marked as poor-quality. This is similar to the geriatrics ward (3.2%) in the Belgian study where inpatients were screened for AF (Desteghe *et al* 2017). However, the cardiology ward in the Belgian study and the German and Danish studies investigating AF screening in patients demonstrated 6.6%, 13% and 20% of their ECGs marked as poor-quality respectively (Desteghe *et al* 2017, Poulsen *et al* 2017, Wegner *et al* 2020). In these studies, the ECGs were all independently reviewed with either electrophysiologists (Desteghe *et al* 2017, Wegner *et al* 2020) or cardiologists (Poulsen *et al* 2017) who manually classified the ECGs as poor-quality, unlike our study. The STROKESTOP trial used the same algorithm as our study and classified 0.99% of ECGs as poor-quality (Svennberg *et al* 2017). In SAFER, a higher proportion of ECGs were deemed poor quality than in STROKESTOP (3.7% vs 0.99%). SAFER comprises a different population (no upper age-limit), and no face-to-face training was carried out. In contrast, STROKESTOP participants were all aged 75 or 76, and received face-to-face training in screening centres (Svennberg *et al* 2017).

### 4.3. Strengths and limitations

This is the first study to explore the use of telephone calls as an intervention to improve ECG quality. The inclusion of a control group led to the finding that ECG quality improved over time in both those who received training and those who did not. We checked that this was not explained by a tendency of outliers toward the mean (*i.e.* subjects with an outlying proportion of poor-quality ECGs early in screening tending towards the mean proportion later in screening), adding confidence to our observation that ECG quality improved over time (Bland and Altman 1994).

However, allocation to the telephone intervention was not random but dependent upon team capacity, which meant that it was prone to selection bias. Team capacity issues are likely to explain the small decline in quality of ECGs moving from the remote feasibility study to the Trial, when many more participants were being screened and a lower proportion of participants meeting the threshold for a training call actually received a training call. Secondly, this study was performed using only one handheld ECG device (Zenicor One, Zenicor Medical Systems AB, Sweden) and other methods of recording single-lead ECGs are available (Poulsen *et al* 2017). Studies using the most widely used alternative ECG device (Alivecor) have not reported ECG quality to our knowledge (Godin *et al* 2019, Ko and Jeong 2021). We also combined the remote feasibility study and Trial datasets for our analysis. We felt that differences in methodology were minimal and therefore it was reasonable to combine the data in a single analysis. For instance, in both studies, participants recorded four ECGs per day for three weeks using one particular device, the same criteria were used to identify participants who may benefit from telephone training, and the same training procedure was used.

### 4.4. Implications

**1. Adequate quality ECGs are obtained from remote screening**

We found that the majority of ECGs obtained remotely using the Zenicor One device are of adequate quality. Nonetheless, future research into the use of telehealth ECGs should continue to assess the quality of ECGs, as these results may not generalise to other devices or patient populations.

**2. A minimum time period of screening is required to allow for natural improvements in quality**

This study has highlighted that the quality of ECGs improved over time regardless of whether or not participants received training. This is likely to reflect increasing familiarity with and confidence in using the device over time. An implication of this is that screening should be carried out over a minimum time period to benefit from this natural improvement. We suggest at least two weeks of screening (preferably three) is required.

**3. The role of real-time monitoring of ECG quality**

In the SAFER Programme, the quality of ECGs was monitored in near real-time allowing additional training to be provided to those participants with high numbers of poor-quality ECGs. However, this study did not find a significant benefit to telephone training to improve ECG quality beyond the natural improvements which occur over time without training. Therefore, telephone training might be deemed unnecessary in AF screening, given that it can be labour-intensive and there was sufficient improvement in ECG quality in most participants without any intervention. However, real-time monitoring of ECG quality might still be beneficial for early identification of malfunctioning devices.

## 5. Conclusion

Most participants of ECG-based AF screening studies achieved sufficiently high levels of adequate quality ECGs. For the minority that did not, ECG quality improved over time regardless of whether or not they received additional telephone training. Telephone training did not result in greater improvement in the quality of ECGs over time compared to the natural improvement amongst those who did not receive telephone training. These findings indicate that telephone training may not be required to achieve improvements in ECG quality during screening.

## Data Availability

Requests for pseudonymised data should be directed to the SAFER study co-ordinator (Andrew Dymond using SAFER@medschl.cam.ac.uk) and will be considered by the investigators, in accordance with participant consent. The code used in this analysis is available at https://github.com/peterhcharlton/pq_call_analysis (and archived at https://zenodo.org/doi/10.5281/zenodo.13343455). All data that support the findings of this study are included within the article (and any supplementary information files).
